# Compliance Trajectory and Patterns of COVID-19 Preventive Measures, Japan, 2020–2022

**DOI:** 10.3201/eid2909.221754

**Published:** 2023-09

**Authors:** Taro Kusama, Kenji Takeuchi, Yudai Tamada, Sakura Kiuchi, Ken Osaka, Takahiro Tabuchi

**Affiliations:** Tohoku University Graduate School of Dentistry, Sendai, Japan (T. Kusama, K. Takeuchi, Y. Tamada, S. Kiuchi, K. Osaka);; Nagoya University Graduate School of Medicine, Nagoya, Japan (K. Takeuchi, Y. Tamada);; Tohoku University Frontier Research Institute for Interdisciplinary Sciences, Sendai (S. Kiuchi);; Osaka International Cancer Institute, Osaka, Japan (T. Tabuchi);; The Tokyo Foundation for Policy Research, Tokyo, Japan (T. Tabuchi)

**Keywords:** COVID-19, respiratory infections, severe acute respiratory syndrome coronavirus 2, SARS-CoV-2, SARS, coronavirus disease, coronavirus, preventive measures, sanitation, ventilation, physical distancing, longitudinal studies, viruses, zoonoses, Japan

## Abstract

COVID-19 remains a global health threat. Compliance with nonpharmaceutical interventions is essential because of limited effectiveness of COVID-19 vaccines, emergence of highly contagious variants, and declining COVID-19 antibody titers over time. We evaluated compliance with 14 nonpharmaceutical intervention–related COVID-19 preventive behaviors, including mask wearing, ventilation, and surface sanitation, in a longitudinal study in Japan using 4 waves of Internet survey data obtained during 2020–2022. Compliance with most preventive behaviors increased or remained stable during the 2-year period, except for surface sanitation and going out behaviors; compliance with ventilation behavior substantially decreased in winter. Compliance patterns identified from latent class analysis showed that the number of persons in the low compliance class decreased, whereas those in the personal hygiene class increased. Our findings reflect the relaxation of mobility restriction policy in Japan, where the COVID-19 pandemic continues. Policymakers should consider behavioral changes caused by new policies to improve COVID-19 prevention strategies.

COVID-19, caused by SARS-CoV-2, remains a global health threat (*1*). Although COVID-19 vaccinations have covered most of the population in many countries (*2*), vaccine effectiveness is limited because of the emergence of highly contagious variants and a decrease in SARS-CoV-2 antibody titer over time (*3*,*4*). Therefore, individual compliance with nonpharmaceutical interventions (NPIs), such as physical distancing, mask wearing, and increased building ventilation, remains essential for COVID-19 prevention (*5*–*7*).

Temporal changes in compliance with several NPI-related preventive behaviors have been reported (*8*–*11*). However, those studies were followed up for a short period (e.g., within 0.5–1 year) and included limited types of preventive behaviors. Since the first case of COVID-19 was confirmed, some preventive measures, such as surface sanitation, have been considered less effective, whereas others, such as increased building ventilation, have been confirmed as more effective in general settings (*12*,*13*). Previous studies have not examined changes in preventive measure compliance over time. A cross-sectional study reported patterns of compliance with multiple preventive measures (*14*); however, whether those compliance patterns changed over time remained unclear. We performed a 2-year longitudinal study in Japan to determine changes in compliance with 14 NPI-related COVID-19 preventive behaviors; identify compliance patterns for those behaviors over time; and define sociodemographic characteristics associated with compliance for each preventive behavior and characteristics associated with compliance patterns for multiple preventive behaviors.

## Methods

### Study Design and Participants

We conducted a 2-year follow-up longitudinal study by using unbalanced panel data obtained from 2 Japan COVID-19 and Society Internet Surveys (JACSIS) and 2 Japan Society and New Tobacco Internet Surveys (JASTIS). JACSIS aimed to evaluate health conditions and social determinants during the COVID-19 pandemic in Japan, whereas JASTIS aimed to evaluate the status of new tobacco products and their related factors in Japan (*11*,*15*). Surveys were administered via Internet questionnaires. The surveys were conducted during the following periods: August 25–September 30, 2020 (JACSIS2020); February 8–26, 2021 (JASTIS2021); September 27–October 29, 2021 (JACSIS2021); and February 1–28, 2022 (JASTIS2022). Daily numbers of newly confirmed COVID-19 cases in Japan were determined during the survey periods (https://www.mhlw.go.jp/stf/covid-19/open-data.html) (Appendix Figure 1). The original population of all 4 surveys came from the same panel data. Candidates registered as panelists at an Internet research company (Rakuten Insight, https://insight.rakuten.com) in Japan and responded to multiple surveys; therefore, the number of survey waves encountered by participants ranged from 1 to 4. We excluded participants who provided inconsistent or unreliable responses in the questionnaires (e.g., selected applicable for all questions regarding various types of current drug use or chronic diseases, including major noncommunicable diseases) from the analysis. In addition, we excluded participants who were <20 or >79 years of age.

### Outcome Variables

The evaluated outcomes in each survey were compliance with COVID-19 preventive behaviors. We selected 14 preventive behaviors related to COVID-19 NPIs: mask wearing, ventilation, social distancing, avoiding crowds, hand sanitation, hand washing, gargling, respiratory hygiene, avoiding touching one’s face, surface sanitation, avoiding travel, avoiding going out, avoiding talking closely, and avoiding meeting high-risk persons. We asked the participants about their compliance with each of those preventive behaviors; participants who answered that they always complied were considered compliant with each preventive behavior (Appendix Table 1). Mask wearing, ventilation, social distancing, and avoiding crowds were behaviors mandated by the government of Japan campaign called the 3 Cs, which requests that the public should avoid closed spaces, crowded places, and close-contact settings to prevent COVID-19 (*1*,*16*).

### Predictors

For predictors, we used a continuous scale for survey waves (recorded as 0 for JACSIS2020, 1 for JASTIS2021, 2 for JACSIS2021, and 3 for JASTIS2022), survey type (JACSIS or JASTIS), sex, age categories, education, and equivalent income. For age, education, and equivalent income, we used the values from each survey. Surveys were conducted in the summer/autumn (JACSIS) and winter (JASTIS). In addition, we included population density at the prefecture level as a geographic predictor as a binary variable categorized as the top 20% of densely populated prefectures in Japan, which are Tokyo, Osaka, Kanagawa, Saitama, Aichi, Chiba, Fukuoka, Okinawa, and Hyogo.

### Statistical Analysis

We estimated the absolute differences in percentages and 95% CIs for each preventive behavior according to generalized estimating equations, fitting the Gaussian distribution and identity link function by using a Huber–White sandwich estimator for SEs (*17*). We identified compliance patterns for multiple preventive behaviors to simplify interpretation and gain a holistic understanding of preventive behavior compliance during the COVID-19 pandemic and used latent class analysis to identify those patterns (*18*). We estimated the probability of being in each class on the basis of the generalized structural equation model fitting the logistic regression model and included the binary variables of preventive behavior compliance as dependent variables. We determined the final number of latent classes according to a scree plot of the Bayesian information criterion and proportion of participants belonging to the smallest class. For the scree plot, we estimated the Bayesian information criterion for each model by using a different number of latent classes from 1 to 6; the elbow of the scree plot was considered to have an appropriate number of classes (*18*). Furthermore, a class representing a small portion of the population would violate the generalizability and interpretability of the result; therefore, we excluded the model that estimated >1 class that included <15% of participants (*18*). To avoid violating the local independence assumption within the class, we excluded the preventive behavior that had a ϕ coefficient of >0.7 with the other behaviors (*18*). We found a strong correlation between social distancing and avoiding talking closely behaviors (Appendix Table 2); therefore, we excluded the avoiding talking closely behavior from latent class analysis. 

We also used the estimated class as the outcome and evaluated its association with predictors. We fitted the multinomial logistic regression model with generalized estimating equations and estimated the absolute difference in probability (percentage and 95% CIs) of belonging to each class according to each predictor by using the parametric g-formula (*19*). To evaluate the mobility of latent classes through the 4 surveys, we created a Sankey plot of the proportion of each class at each survey point for participants who responded to all 4 survey waves. To reduce selection bias, in all statistical analyses, including descriptive statistics and regression analysis, we used the inverse probability weighting method and propensity score estimated from the Comprehensive Survey of Living Conditions, which is representative of a sociodemographic random sample in Japan (*20*). We used generalized estimating equations for data with multiple responses among persons and addressed interindividual correlations; therefore, the results obtained from regression analysis can be interpreted as a population-average difference in preventive behavior compliance (*21*). Only the candidates who completed the whole questionnaire could register their responses within the online system created by the Internet research company; no missing values existed for any participant in this study. We used Stata version 17.0 (StataCorp LLC, https://www.stata.com) for all analyses and set statistical significance at α = 0.05.

### Ethical Issues

Both the JACSIS and JASTIS conducted during 2020–2022 followed procedures approved by the Ethics Committee on Research of Human Subjects at the Osaka International Cancer Institute (no. 20084-8). In addition, we followed Strengthening the Reporting of Observational studies in Epidemiology guidelines, known as STROBE, to report our observational study.

## Results

Initially, the numbers of responses to the questionnaires were 28,000 for JACSIS2020, 26,000 for JASTIS2021, 31,000 for JACSIS2021, and 33,000 for JASTIS2022. After excluding respondents who did not meet eligibility criteria, we included 103,312 responses from a total of 41,510 participants (Table 1; Appendix Figure 2) and determined response patterns and distribution (Appendix Table 3). Characteristics of the respondents were recorded; the average age (±SD) of participants was 47.2 ±17.3 SD years; 49.9% were men and 50.1% women (Table 2).

**Table 1:** Preventive behaviors of participants in each survey in study of compliance trajectory and patterns of COVID-19 preventive measures, Japan, 2020–2022*

Characteristics	Compliance	Surveys				
		All responses	JACSIS2020	JASTIS2021	JACSIS2021	JASTIS2022
No. responses	NA	103,312 (100.0)	24,651 (100.0)	22,350 (100.0)	27,348 (100.0)	28,963 (100.0)
Preventive behaviors						
Mask-wearing	Yes	91,377 (88.5)	20,624 (83.7)	19,415 (86.9)	24,930 (91.2)	26,407 (91.2)
	No	11,935 (11.5)	4,027 (16.3)	2,935 (13.1)	2,418 (8.8)	2,556 (8.8)
Ventilation	Yes	42,240 (40.9)	11,221 (45.5)	8,066 (36.1)	13,464 (49.2)	9,489 (32.8)
	No	61,072 (59.1)	13,430 (54.5)	14,284 (63.9)	13,884 (50.8)	19,474 (67.2)
Social distancing	Yes	45,759 (44.3)	10,317 (41.8)	9,884 (44.2)	12,855 (47.0)	12,703 (43.9)
	No	57,553 (55.7)	14,334 (58.2)	12,466 (55.8)	14,493 (53.0)	16,260 (56.1)
Avoiding crowds	Yes	61,679 (59.7)	14,771 (59.9)	13,216 (59.1)	17,092 (62.5)	16,600 (57.3)
	No	41,633 (40.3)	9,880 (40.1)	9,134 (40.9)	10,256 (37.5)	12,363 (42.7)
Hand sanitation	Yes	68,435 (66.2)	14,550 (59.0)	14,696 (65.8)	19,034 (69.6)	20,155 (69.6)
	No	34,877 (33.8)	10,101 (41.0)	7,654 (34.2)	8,314 (30.4)	8,808 (30.4)
Handwashing	Yes	57,316 (55.5)	13,551 (55.0)	11,962 (53.5)	15,986 (58.5)	15,817 (54.6)
	No	45,996 (44.5)	11,100 (45.0)	10,388 (46.5)	11,362 (41.5)	13,146 (45.4)
Gargling	Yes	47,298 (45.8)	10,859 (44.0)	10,736 (48.0)	12,373 (45.2)	13,330 (46.0)
	No	56,014 (54.2)	13,792 (56.0)	11,614 (52.0)	14,975 (54.8)	15,633 (54.0)
Respiratory hygiene	Yes	75,845 (73.4)	16,817 (68.2)	15,810 (70.7)	20,967 (76.7)	22,250 (76.8)
	No	27,467 (26.6)	7,834 (31.8)	6,540 (29.3)	6,381 (23.3)	6,713 (23.2)
Avoiding touching face	Yes	45,945 (44.5)	10,633 (43.1)	9,707 (43.4)	12,451 (45.5)	13,154 (45.4)
	No	57,367 (55.5)	14,018 (56.9)	12,643 (56.6)	14,897 (54.5)	15,809 (54.6)
Surface sanitation	Yes	21,378 (20.7)	5,101 (20.7)	4,880 (21.8)	5,648 (20.6)	5,748 (19.8)
	No	81,935 (79.)3	19,550 (79.3)	17,470 (78.2)	21,700 (79.4)	23,215 (80.2)
Avoiding travel	Yes	74,478 (72.1)	17,323 (70.3)	16,542 (74.0)	20,571 (75.2)	20,042 ()69.2
	No	28,834 (27.9)	7,328 (29.7)	5,808 (26.0)	6,777 (24.8)	8,921 (30.8)
Avoiding going out	Yes	58,274 (56.4)	14,648 (59.4)	12,839 (57.4)	15,726 (57.5)	15,061 (52.0)
	No	45,038 (43.6)	10,003 (40.6)	9,511 (42.6)	11,622 (42.5)	13,902 (48.0)
Avoiding talking closely	Yes	43,499 (42.1)	9,796 (39.7)	9,160 (41.0)	12,266 (44.8)	12,277 (42.4)
	No	59,813 (57.9)	14,855 (60.3)	13,190 (59.0)	15,082 (55.2)	16,686 (57.6)
Avoiding meeting persons at high risk	Yes	61,173 (59.2)	14,402 (58.4)	12,339 (55.2)	17,405 (63.6)	17,028 (58.8)
	No	42,139 (40.8)	10,249 (41.6)	10,011 (44.8)	9,943 ()36.4	11,935 (41.2)

*Values are no. (%) of responses to questionnaires. JACSIS, Japan COVID-19 and Society Internet Survey (2020, 2021); JASTIS, Japan Society and New Tobacco Internet Survey (2021, 2022).

**Table 2:** Sociodemographic characteristics of participants in each survey in study of compliance trajectory and patterns of COVID-19 preventive measures, Japan, 2020–2022*

Characteristics	Surveys				
	All responses	JACSIS2020	JASTIS2021	JACSIS2021	JASTIS2022
No. responses	103,312	24,651	22,350	27,348	28,963
Sex					
M	51,540 (49.9)	12,422 (50.4)	11,467 (51.3)	13,473 (49.3)	14,179 (49.0)
F	51,772 (50.1)	12,229 (49.6)	10,882 (48.7)	13,875 (50.7)	14,784 (51.0)
Age, y					
20–29	15,650 (15.1)	3,323 (13.5)	2,816 (12.6)	3,544 (13.0)	5,967 (20.6)
30–39	15,158 (14.7)	3,794 (15.4)	3,208 (14.4)	4,165 (15.2)	3,990 (13.8)
40–49	20,151 (19.5)	4,954 (20.1)	4,467 (20.0)	5,446 (19.9)	5,284 (18.2)
50–59	17,928 (17.3)	4,283 (17.4)	4,245 (19.0)	4,794 ()17.5	4,606 (15.9)
60–69	18,033 (17.5)	4,290 (17.4)	4,185 (18.7)	4,844 (17.7)	4,715 (16.3)
70–79	16,392 (15.9)	4,007 (16.2)	3,429 (15.3)	4,555 (16.7)	4,401 (15.2)
Education					
Junior high school, high school	50,397 (48.8)	11,639 (47.2)	11,026 (49.4)	13,602 (49.7)	14,130 (48.8)
Vocational school, junior college	20,820 (20.2)	4,962 (20.1)	4,365 (19.5)	5,576 (20.4)	5,917 (20.4)
University, graduate school	31,341 (30.3)	7,908 (32.1)	6,887 (30.8)	7,930 (29.0)	8,617 (29.8)
Other	754 (0.7)	142 (0.6)	72 (0.3)	240 (0.9)	299 (1.0)
Equivalent income, million JPY					
<2.00	18,261 (17.7)	4,388 (17.8)	3,994 (17.9)	4,743 (17.3)	5,136 (17.7)
2.00–3.99	37,976 (36.8)	9,235 (37.5)	8,422 (37.7)	9,953 (36.4)	10,366 (35.8)
4.00–5.99	14,305 (13.8)	3,183 (12.9)	3,103 (13.9)	3,894 (14.2)	4,125 (14.2)
>6.00	9,741 (9.4)	2,673 (10.8)	2,036 (9.1)	2,428 (8.9)	2,604 (9.0)
Not answered	23,029 (22.3)	5,172 (21.0)	4,795 (21.5)	6,330 (23.2)	6,732 (23.3)
Population density of residential prefecture					
High, top 20%	30,580 (29.6)	7,413 (30.1)	6,422 (28.7)	8,296 (30.3)	8,448 (29.2)
Low, <80%	72,732 (70.4)	17,238 (69.9)	15,928 (71.3)	19,052 (69.7)	20,515 (70.8)

*Values are no. (%) of responses to questionnaires. JACSIS, Japan COVID-19 and Society Internet Survey (2020, 2021); JASTIS, Japan Society and New Tobacco Internet Survey (2021, 2022); JPY, Japanese yen.

**Figure 1:**
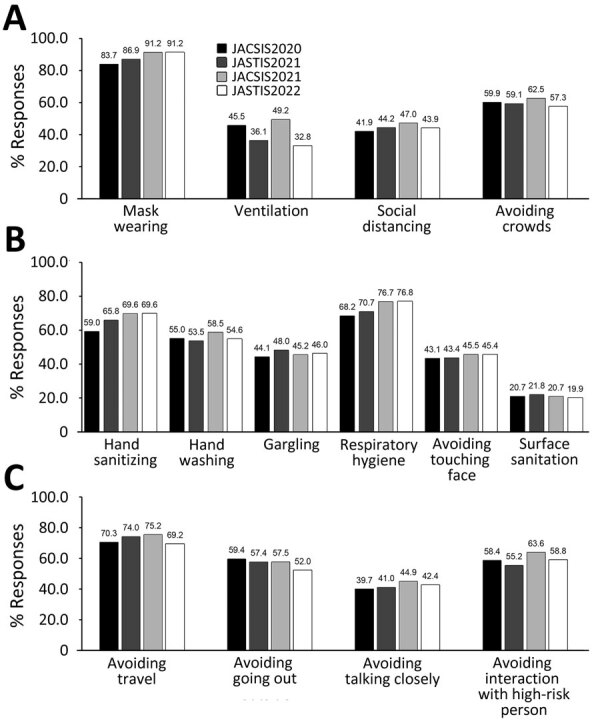
Transition of compliance with COVID-19 preventive behaviors over time in study of compliance trajectory and patterns of COVID-19 preventive measures, Japan, 2020–2022. Four surveys were conducted during August 25–September 30, 2020 (JACSIS2020); February 8–26, 2021 (JASTIS2021); September 27–October 29, 2021 (JACSIS2021); and February 1–28, 2022 (JASTIS2022). Percentages of responses from study participants indicate compliance with behaviors related to 3 behavioral areas: A) 3 Cs, a government of Japan campaign mandating the public to avoid closed spaces, crowded places, and close-contact settings to prevent COVID-19; B) personal hygiene; and C) avoiding social contact. Number of questionnaire responses was 103,312 from a total of 41,510 study participants. Values above bars indicate specific percentages. JACSIS, Japan COVID-19 and Society Internet Survey; JASTIS, Japan Society and New Tobacco Internet Survey.

We evaluated compliance with each preventive behavior according to the survey period (Figure 1). Compliance with most behaviors slightly increased or remained stable among the surveys; however, compliance with ventilation and avoiding going out behaviors decreased among the surveys. Compliance with ventilation showed apparent seasonal fluctuations. We reported the characteristics of participants who complied with each preventive behavior (Appendix Table 4) and estimated the associations among participant characteristics and each preventive behavior by using a multivariable regression model (Table 3; Appendix Tables 5–7). Compliance with most preventive behaviors did not significantly decrease among the survey waves, except for the surface sanitation and avoiding going out behaviors. Ventilation compliance decreased by 13.4% (95% CI −14.4% to −12.3%) for JASTIS (winter season). For all preventive behaviors, compliance was significantly higher among women than men. Older age, higher education, and higher income (i.e., incremental increases of each variable) were associated with greater compliance with most preventive behaviors. Compliance with COVID-19 preventive behaviors differed according to population density of residential prefectures; however, the direction of associations differed depending on the preventive behavior.

**Table 3:** Trends in associations between preventive behaviors and participant characteristics (n = 82,201 responses) in study of compliance trajectory and patterns of COVID-19 preventive measures, Japan, 2020–2022*

Preventive behavior	Per wave†	JASTIS‡	Women§	Older age¶	Higher education#	Higher equivalent income**	High population density††
Mask wearing	+	–	+ + +	+	NS	+	NS
Ventilation	NS	– – –	+ + +	+	+	NS	+
Social distancing	+	−	+ +	+	+	NS	+
Avoiding crowds	NS	−	+ + +	+	+	NS	+
Hand sanitation	+	NS	+ + +	NS	NS	+	NS
Hand washing	+	–	+ + +	NS	NS	+	NS
Gargling	NS	+	+ +	NS	+	NS	+ +
Respiratory hygiene	+	–	+ + +	NS	+	+	–
Avoiding touching face	+	NS	+ + +	+	+	+	+
Surface sanitation	–	+	+ +	–	NS	+	NS
Avoiding travel	NS	NS	+ + +	+	–	–	–
Avoiding going out	–	–	+ + +	+	+	–	–
Avoiding talking closely	+	–	+ +	+	+	NS	+
Avoiding high-risk person	+	–	+ + +	+	NS	NS	NS

*p value for trend was <0.05. +, 0%–5% difference; + +, >5%–10% difference; + + +, >10% difference; –, indicates >–5% to 0% difference; – –, indicates –10% to –5% difference; – – –, indicates <–10% difference. Differences were estimated by increments within each category. JACSIS, Japan COVID-19 and Society Internet Survey; JASTIS, Japan Society and New Tobacco Internet Survey; NS, not significant. †Surveys were conducted in 4 waves: August 25–September 30, 2020 (JACSIS2020); February 8–26, 2021 (JASTIS2021); September 27–October 29, 2021 (JACSIS2021); and February 1–28, 2022 (JASTIS2022). ‡Referent was JACSIS. §Referent was men. ¶Age was treated as a continuous variable (i.e., 20–29 y = 1, 30–39 y = 2, 40–49 y = 3, 50–59 y = 4, 60–69 = 5, 70–79 = 6); associations were estimated according to an incremental increase in age. #Participants who answered as other for education were excluded. Education was treated as a continuous variable (i.e., junior high school, high school = 1, vocational school, junior college = 2, university, graduate school = 3); associations were estimated according to an incremental increase in education level. **Participants who did not answer income question were excluded. Equivalent income (million yen) was treated as a continuous variable (i.e., <2.00 = 1, 2.00–3.99 = 2, 4.00–5.99 = 3, >6.00 = 4); associations were estimated according to an incremental increase in income. ††Top 20% of residential prefecture population density. Referent was low (<80%) density.

**Figure 2:**
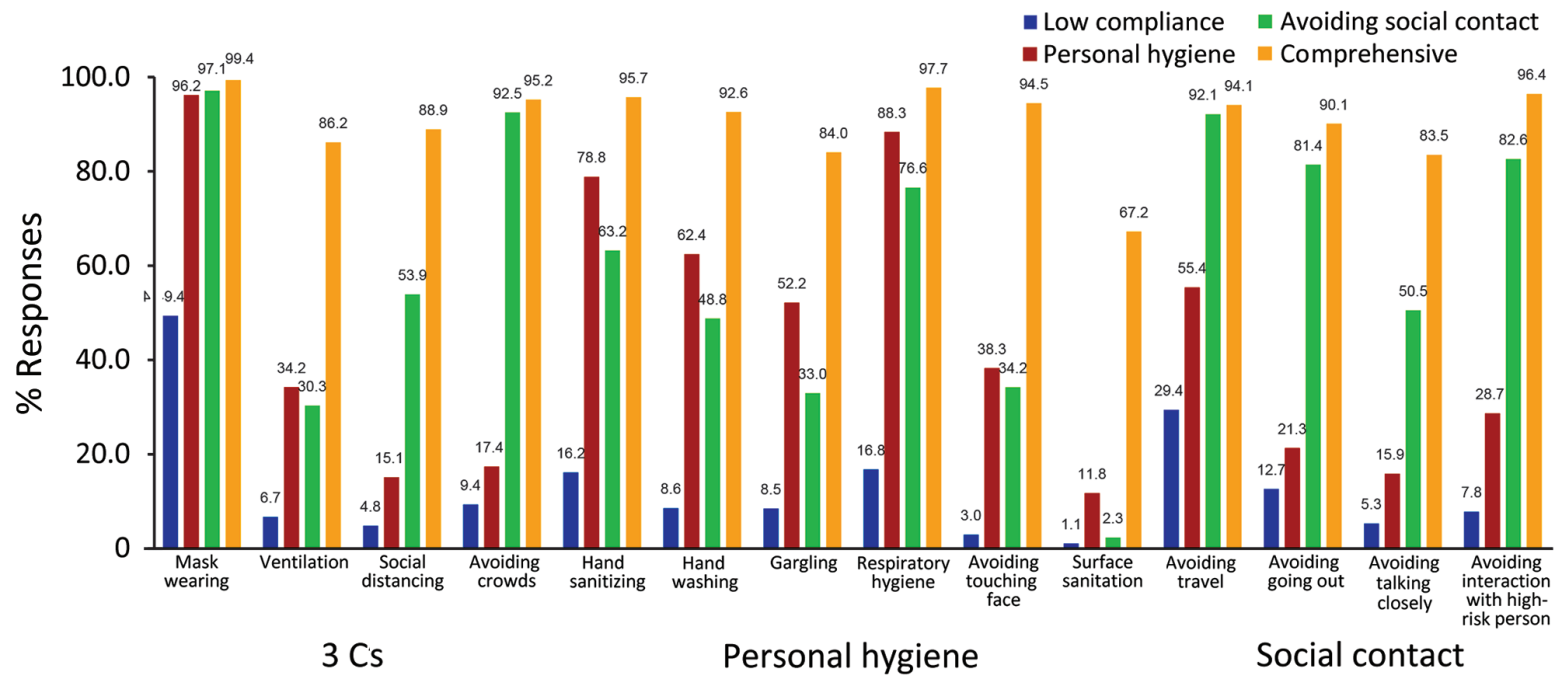
Compliance with each preventive behavior according to 4 latent classes in study of compliance trajectory and patterns of COVID-19 preventive measures, Japan, 2020–2022. Percentage of responses to questions regarding each behavior is shown; 3 Cs is a government of Japan campaign mandating the public to avoid closed spaces, crowded places, and close-contact settings to prevent COVID-19. Values above bars indicate specific percentages.

**Figure 3:**
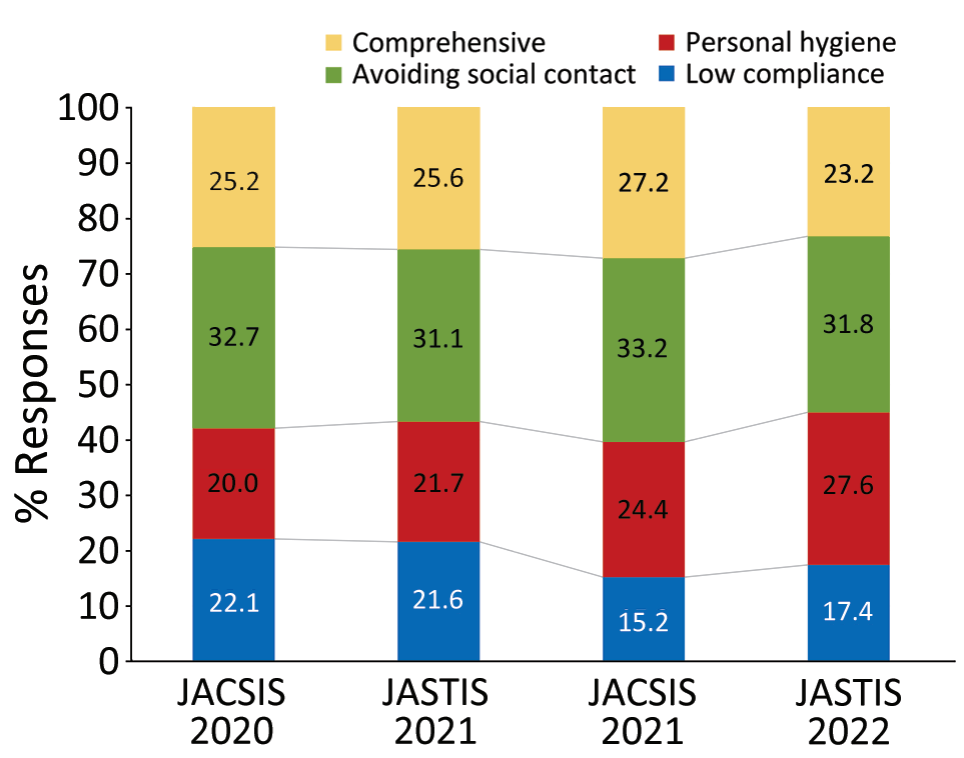
Percentage of persons belonging to each of 4 latent classes according to survey waves (n = 103,312 responses) in study of compliance trajectory and patterns of COVID-19 preventive measures, Japan, 2020–2022. Four surveys were conducted during August 25–September 30, 2020 (JACSIS2020); February 8–26, 2021 (JASTIS2021); September 27–October 29, 2021 (JACSIS2021); and February 1–28, 2022 (JASTIS2022). Values within bar sections indicate specific percentages. JACSIS, Japan COVID-19 and Society Internet Survey; JASTIS, Japan Society and New Tobacco Internet Survey.

We determined that the number of latent classes was 4 according to the scree plot, distribution of class allocation, and interpretability (Appendix Figure 3). We evaluated the distribution of compliance with each preventive behavior for the 4 identified classes (Figure 2; Appendix Table 8). Class 1 was low compliance, which was characterized by lower than average compliance with all preventive behaviors. Class 2 was personal hygiene, which was characterized by higher than average compliance with personal hygiene measures, such as hand sanitation or respiratory hygiene, and lower than average compliance with the other measures. Class 3 was avoiding social contact, which was characterized by higher than average compliance with measures related to social contacts, such as avoiding travel or avoiding crowds, whereas compliance with other measures was similar to the overall average. Class 4 was comprehensive, which was characterized by higher than average compliance with all measures within the other classes. The percentage of persons in the low compliance class decreased over time, whereas the percentage in the personal hygiene class increased (Figure 3). We categorized the characteristics of the participants belonging to each latent class (Appendix Table 9). Using the multinomial logistic regression model, we estimated associations between participant characteristics and latent classes by determining percentage differences and odds ratios (Table 4; Appendix Table 10). The percentage of persons in the low compliance class significantly decreased (−2.8% [95% CI −3.3% to −2.3%] per wave; p<0.001) with each survey wave, whereas those in the personal hygiene class significantly increased (2.6% [95% CI 2.1%–3.0%] per wave; p<0.001). Women were less likely to belong to the low compliance class than men (−15.8% [95% CI −17.0% to −14.7%]). Furthermore, younger participants tended to belong to the low compliance or personal hygiene class, whereas those who were older tended to be categorized into the avoiding social contact class. In addition, those with lower education tended to be allocated to the low compliance class; those with higher education tended to belong to the comprehensive class. Participants with lower income tended to be allocated to the low compliance or avoiding social contact class, whereas those with higher income tended to be categorized into the personal hygiene or comprehensive class. Participants who lived in highly populated prefectures were less likely to belong to the avoiding social contact class and more likely to be in the comprehensive class.

**Table 4:** Associations (% difference) between each latent class and participant characteristic in study of compliance trajectory and patterns of COVID-19 preventive measures, Japan, 2020–2022*

Characteristic	Low compliance			Personal hygiene			Avoiding social contact			Comprehensive	
	% Diff (95% CI)	p value		% Diff (95% CI)	p value		% Diff (95% CI)	p value		% Diff (95% CI)	p value
Per wave†	–2.8 (–3.3 to −2.3)	<0.001		2.6 (2.1–3.0)	<0.001		0.4 (–0.1 to 1.0)	0.098		−0.2 (–0.7 to 0.3)	0.482
Survey											
JACSIS	Referent	NA		Referent	NA		Referent	NA		Referent	NA
JASTIS	3.4 (2.6–4.2)	<0.001		–0.2 (–1.1 to 0.7)	0.618		–1.5 (–2.6 to –0.4)	0.008		–1.7 (–2.6 to –0.8)	<0.001
Sex											
M	Referent	NA		Referent	NA		Referent	NA		Referent	NA
F	–15.8 (–17.0 to –14.7)	<0.001		2.1 (1.0–3.3)	<0.001		2.8 (1.4–4.3)	<0.001		10.7 (9.2–12.3)	<0.001
Age, y											
20–29	Referent	NA		Referent	NA		Referent	NA		Referent	NA
30–39	–7.8 (–10.5 to –5.2)	<0.001		–1.0 (–3.3 to 1.3)	0.392		6.9 (4.9–8.8)	<0.001		2.0 (–0.1 to 4.0)	0.052
40–49	–10.7 (–13.3 to –8.0)	<0.001		–0.9 (–3.2 to 1.3)	0.402		8.5 (6.7–10.3)	<0.001		3.1 (1.2–5.1)	0.002
50–59	–12.2 (–14.9 to –9.5)	<0.001		–1.6 (–3.9 to 0.8)	0.189		12.2 (10.3–14.0)	<0.001		1.6 (–0.4 to 3.5)	0.113
60–69	–13.4 (–16.1 to –10.8)	<0.001		–3.8 (–6.1 to –1.5)	0.001		16.9 (14.8–19.0)	<0.001		0.3 (–1.8 to 2.4)	0.757
70–79	–15.6 (–18.4 to –12.8)	<0.001		–9.7 (–12.4 to –7.1)	<0.001		22.8 (19.5–26.1)	<0.001		2.5 (–1.0 to 6.0)	0.160
Education											
Junior high, high school	Referent	NA		Referent	NA		Referent	NA		Referent	NA
Vocational school, junior college	–4.1 (–5.3 to –2.8)	<0.001		0.1 (–1.2 to 1.3)	0.916		–0.6 (–2.0 to 0.7)	0.356		4.6 (3.3–6.0)	<0.001
University, graduate school	–2.3 (–3.7 to –1.0)	0.001		–0.5 (–1.9 to 0.9)	0.460		–1.0 (–2.9 to 0.9)	0.300		3.8 (1.8–5.8)	<0.001
Equivalent income, million JPY	2.7 (–3.6 to 9.1)	0.404		–3.7 (–10.8 to 3.4)	0.307		–2.4 (–10.3 to 5.6)	0.556		3.4 (–2.8 to 9.6)	0.284
<2.00	Referent	NA		Referent	NA		Referent	NA		Referent	NA
2.00–3.99	–3.4 (–5.1 to –1.6)	<0.001		4.2 (2.7–5.7)	<0.001		–0.9 (–2.9 to 1.0)	0.343		0.2 (–2.0 to 2.3)	0.889
4.00–5.99	–4.4 (–6.3 to –2.4)	<0.001		5.0 (3.3–6.7)	<0.001		–2.2 (–4.3 to –0.1)	0.040		1.6 (–0.6 to 3.8)	0.159
>6.00	–3.4 [–6.3 to –0.4)	0.024		6.6 (3.9–9.4)	<0.001		–6.5 (–8.8 to –4.2)	<0.001		3.2 (0.5–6.0)	0.022
Not answered	–2.7 (–4.6 to –0.8)	0.005		0.5 (–1.2 to 2.2)	0.560		–1.6 (–4.0 to 0.7)	0.179		3.8 (1.5–6.2)	0.001
Population density of residential prefecture											
Low	Referent	NA		Referent	NA		Referent	NA		Referent	NA
High	–0.3 (–1.5 to 1.0)	0.685		1.3 (0.1–2.5)	0.041		–4.1 (–5.6 to –2.7)	<0.001		3.1 (1.6–4.6)	<0.001

*Total number of responses was 103,312. Diff, difference; JACSIS, Japan COVID-19 and Society Internet Survey; JASTIS, Japan Society and New Tobacco Internet Survey; JPY, Japanese yen; NA, not applicable. †Surveys were conducted in 4 waves: August 25–September 30, 2020 (JACSIS2020); February 8–26, 2021 (JASTIS2021); September 27–October 29, 2021 (JACSIS2021); and February 1–28, 2022 (JASTIS2022).

**Figure 4:**
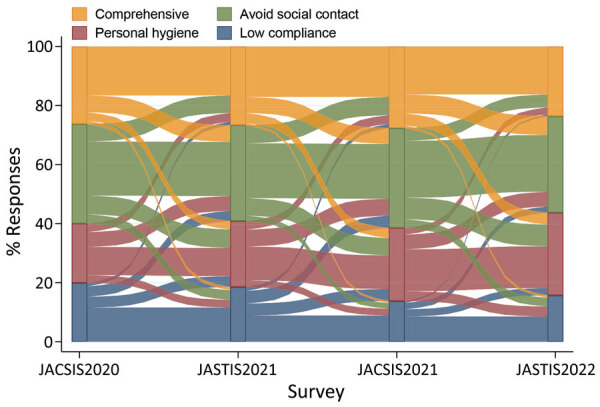
Compliance patterns for survey participants who responded to all 4 surveys waves (11,804 participants) in study of compliance trajectory and patterns of COVID-19 preventive measures, Japan, 2020–2022. Four surveys were conducted during August 25–September 30, 2020 (JACSIS2020); February 8–26, 2021 (JASTIS2021); September 27–October 29, 2021 (JACSIS2021); and February 1–28, 2022 (JASTIS2022). The Sankey plot shows compliance patterns of persons in 4 latent classes for each survey wave. JACSIS, Japan COVID-19 and Society Internet Survey; JASTIS, Japan Society and New Tobacco Internet Survey.

For participants who completed the 4 survey waves (n = 11,804), we used a Sankey diagram to compare patterns of the 4 latent classes among the 4 survey waves (Figure 4). Although most preventive behavior patterns were consistent, some changed among the 4 survey periods. We observed a large influx of participants into the personal hygiene class from the low compliance and avoiding social contact classes over time.

## Discussion

Our results show that compliance with most COVID-19 preventive behaviors included in this study either increased or remained stable over the 4 survey waves; however, compliance with surface sanitation and avoiding going out behaviors decreased. Compliance with ventilation substantially decreased during the winter seasons. Female sex, older age, higher education, and higher equivalent income were positively associated with compliance with most preventive behaviors. The percentage of persons in the low compliance class decreased over time and increased in the personal hygiene class, which could be partially attributed to the change in the overall pattern toward personal hygiene compliance.

Previous studies reported changes in compliance with COVID-19 preventive behaviors. A 1-year follow-up study conducted in the United States reported that compliance with mask wearing continuously increased from April 2020 to April 2021; however, compliance with physical distancing and reduced movement was stable or decreased slightly over time (*9*). Another study also reported that compliance with mask wearing increased over time, and compliance with physical distancing decreased (*10*). Similarly, our study showed increased compliance with mask wearing; however, although compliance with the avoiding going out behavior decreased, compliance with social distancing increased over time. This result could be because of changes in Japan’s policy that relaxed social distancing rules and promoted travel and going out (*22*). Previous studies have mainly investigated changes in preventive behaviors related to COVID-19 prevention guidelines (e.g., mask wearing and physical distancing) within a relatively short period (<1 year). We studied changes in other preventive behaviors over a 2-year follow-up period, especially noting the seasonal fluctuation of compliance with ventilation and decreased compliance with surface sanitation, avoiding travel, and avoiding going out behaviors.

We showed that women, older and more educated participants, and those with higher income were highly compliant with most preventive behaviors. A previous study also reported that compliance with COVID-19 preventive behaviors was higher among women than men (*23*). Our study showed a large gap in compliance behaviors between men and women. Social desirability bias might have been responsible for those results because compliance was self-reported. However, a previous study suggested that higher self-reported compliance with preventive behaviors reflects actual compliance and is less affected by social desirability bias because of a participant’s sex (*23*). The tendency of older adults to comply with preventive behaviors has also been reported (*24*). Furthermore, socioeconomic disparities have been reported to affect compliance with COVID-19 preventive behaviors, and persons with higher education or income were more compliant with those behaviors (*24*,*25*). Our results also showed that persons with higher education or income were more compliant with all preventive behaviors except for avoiding travel and avoiding going out behaviors. 

Our results revealed differences in compliance with COVID-19 preventive behaviors according to sociodemographic status, including sex, age, education, and income level. Although the government of Japan emphasized the importance of NPIs in preventing COVID-19 through various media sources, such important information might not have reached specific groups, including those with low socioeconomic status. The source of information related to COVID-19 affects preventive behavior compliance in Japan (*11*). For risk communications during a health crisis, the sociodemographic features of groups for which the government attempts to provide essential information should be considered (*26*). In addition, during the initial waves of the COVID-19 pandemic, a severe shortage of surgical and N95 masks existed even in clinical settings (*27**,**28*); therefore, low-income persons would have had difficulty preparing sufficient masks because of the mask shortage and increased cost from reselling. Although the government of Japan provided all citizens with 1 supply of cloth masks (*29*), the distribution and cost of surgical and N95 masks should have been controlled to increase affordability and availability for citizens.

To determine patterns of compliance with multiple preventive behaviors, we identified 4 latent classes on the basis of compliance with each of the 14 preventive behaviors. A previous cross-sectional study identified similar compliance patterns for COVID-19 prevention (*14*). Although that study only considered 6 preventive behaviors, the authors identified a group with low compliance and 1 with high compliance with all preventive behaviors. Moreover, similar to the findings in our study, participants in that study who were included in the low compliance group were predominantly younger, male, and less educated (*14*).

Our study results suggested that the percentage of persons in the low compliance class decreased over time, but the percentage of persons in the personal hygiene class increased. A study in the United Kingdom reported that compliance with COVID-19 prevention guidelines decreased slightly during 1 year (*30*). Although relaxation of mask-wearing rules for vaccinated persons occurred in other countries, including the United States (*31*), the government of Japan did not relax compliance with any preventive behaviors, except for traveling and going out (*22*). Therefore, compliance with the preventive behaviors showed a continuous increase over time in Japan. Moreover, we observed an increased percentage of persons in the personal hygiene class and an influx from the avoiding social contact class to the personal hygiene class. This influx also reflects relaxation of the avoiding social contact policy for COVID-19 prevention (*22*).

The first limitation of our study is possible information bias. Compliance with COVID-19 preventive behaviors was self-reported, and misclassification of responses might have affected our results to some extent. However, as previously described, self-reported compliance is less affected by social desirability bias in both sexes (*23*). In addition, a study using an Internet survey reported that ≈50% of persons did not tell others about their actual compliance with COVID-19 preventive behaviors (*32*). The high level of anonymity of that survey method helped identify actual compliance with preventive behaviors. Therefore, our Internet survey also likely obtained more correct answers from participants than an interview-based survey. A future study using objective measurements for preventive behavior compliance, such as tracking mobile phones, might decrease potential information bias (*33*). The second limitation is selection bias. We recruited participants from the registry of an Internet survey company, and the distribution of characteristics was different from that of the general population in Japan. We calculated a sampling weight by using a representative sample of the population in Japan for analysis in this study; therefore, the representativeness of our results was improved. Furthermore, although some participants did not participate in all 4 survey waves, we could partially eliminate the bias caused by dropout by applying a sampling weight for all 4 waves.

COVID-19 prevention policies varied among nations (*34*), and the magnitude of associations among sociodemographic characteristics and preventive behavior compliance also differed among them (*24*). Therefore, caution should be exercised when generalizing the results of this study to countries outside of Japan.

In conclusion, we conducted a longitudinal follow-up study by using 4 multiple-panel surveys over a 2-year period and showed that compliance with most of the 14 NPI-related COVID-19 preventive behaviors increased or remained stable over time, except for surface sanitation and avoiding going out behaviors; compliance with ventilation decreased during the winter season. Moreover, latent class analysis suggested that compliance patterns changed; the number of persons in the low compliance class decreased over time, whereas the number of persons in the personal hygiene class increased. Overall, compliance with NPI-related COVID-19 preventive behaviors in Japan has increased, which can be partially attributed to changes in compliance patterns among persons. Changes in compliance with NPI-related preventive behaviors in Japan might be because persons prefer to comply with personal hygiene measures under the relaxed mobility restriction policy during the ongoing COVID-19 pandemic. From a public health perspective, policymakers should anticipate potential changes in preventive behavior patterns caused by new policy introduction to improve strategies for future prevention of COVID-19 and other public health threats.

Appendix Additional information for compliance trajectory and patterns of COVID-19 preventive measures, Japan, 2020–2022.
