# Supplementary material for: Compliance Trajectory and Patterns of COVID-19 Preventive Measures, Japan, 2020–2022
Source: Emerg Infect Dis. 2023 Sep;29(9):1747–56. doi: 10.3201/eid2909.221754 (PMC10461672; doi:10.3201/eid2909.221754)
Supplement: Appendix — Additional information for compliance trajectory and patterns of COVID-19 preventive measures, Japan, 2020–2022. [file 22-1754-Techapp-s1.pdf]

Article DOI: https://doi.org/10.3201/eid2909.221754

EID cannot ensure accessibility for supplementary materials supplied by authors. Readers who have difficulty accessing supplementary content should contact the authors for assistance.

Compliance Trajectory and Patterns of COVID-19 Preventive Measures, Japan, 2020–2022

Appendix

Appendix Table 1. Original question asking about compliance with 14 preventive behaviors for COVID-19*

In the past month, how often did you take any of the following preventive measures?		
No.	Preventive behaviors	Original explanation
1	Mask wearing	I wore a mask in a public place.
2	Ventilation	I opened the window to ventilate the room.
3	Social distancing	I tried to maintain social distance (at least 2 m between persons).
4	Avoiding crowds	I tried not to go to places crowded by persons.
5	Hand sanitation	I disinfected my hands and fingers by using rubbing alcohol.
6	Handwashing	I washed my hands with soap for more than 15 s.
7	Gargling	I gargled when I returned home.
8	Respiratory hygiene	I performed cough etiquette.
9	Avoiding touching around the face	I avoided touching my eyes, nose, and mouth with unwashed hands.
10	Surface sanitation	I disinfected doorknobs and other items that are easily touched by one's hands.
11	Avoiding travel	I avoided travel.
12	Avoiding going out	I avoided going out.
13	Avoiding talking closely	I avoided talking or vocalizing at close range (within 1 m).
14	Avoiding high-risk person	I avoided meeting with persons considered to be at high risk for infection.

*Answer choices: 1, always; 2, sometimes; 3, seldom; 4, never.

Appendix Table 2. Phi coefficients showing associations between COVID-19 preventive behaviors*

Behavior	Mask wearing	Ventilation	Social distancing	Avoid crowds	Hand sanitation	Hand washing	Gargling	Respiratory hygiene	Avoid touching face	Surface sanitation	Avoid travel	Avoid going out	Avoid talking closely	Avoid high-risk person
Mask wearing	1.00	NA	NA	NA	NA	NA	NA	NA	NA	NA	NA	NA	NA	NA
Ventilation	0.22	1.00	NA	NA	NA	NA	NA	NA	NA	NA	NA	NA	NA	NA
Social distancing	0.24	0.33	1.00	NA	NA	NA	NA	NA	NA	NA	NA	NA	NA	NA
Avoiding crowds	0.31	0.30	0.49	1.00	NA	NA	NA	NA	NA	NA	NA	NA	NA	NA
Hand sanitation	0.35	0.26	0.26	0.26	1.00	NA	NA	NA	NA	NA	NA	NA	NA	NA
Hand washing	0.27	0.31	0.28	0.28	0.40	1.00	NA	NA	NA	NA	NA	NA	NA	NA
Gargling	0.21	0.30	0.24	0.22	0.28	0.40	1.00	NA	NA	NA	NA	NA	NA	NA
Respiratory hygiene	0.44	0.27	0.26	0.30	0.39	0.35	0.33	1.00	NA	NA	NA	NA	NA	NA
Avoid touching face	0.24	0.38	0.39	0.34	0.33	0.39	0.35	0.37	1.00	NA	NA	NA	NA	NA
Surface sanitation	0.15	0.46	0.33	0.26	0.29	0.31	0.33	0.24	0.45	1.00	NA	NA	NA	NA
Avoid travel	0.38	0.22	0.30	0.40	0.24	0.23	0.19	0.29	0.24	0.17	1.00	NA	NA	NA
Avoid going out	0.29	0.28	0.41	0.52	0.22	0.25	0.21	0.26	0.31	0.26	0.54	1.00	NA	NA
Avoid talking closely	0.24	0.32	0.71	0.47	0.25	0.26	0.23	0.25	0.39	0.34	0.31	0.44	1.00	NA
Avoid high-risk person	0.32	0.31	0.50	0.59	0.30	0.29	0.23	0.34	0.36	0.28	0.38	0.43	0.48	1.00

*Total number of responses was 103,312. NA, not applicable.

Appendix Table 3. Survey response patterns among 41,510 survey participants*

JACSIS2020	JASTIS2021	JACSIS2021	JASTIS2022	No. (%)	No. responses	Total no.	%
X	X	X	X	11,804 (28.4)	4	11,804	28.4
NA	X	X	X	2,283 (5.5)	3	7,390	17.8
X	X	X	NA	2,101 (5.1)			
X	X	NA	X	1,551 (3.7)			
X	NA	X	X	1,455 (3.5)			
NA	NA	X	X	6,629 (16.0)	2	11,610	28.0
X	X	NA	NA	2,661 (6.4)			
X	NA	X	NA	682 (1.6)			
X	NA	NA	X	579 (1.4)			
NA	X	X	NA	559 (1.4)			
NA	X	NA	X	500 (1.2)			
NA	NA	NA	X	4,370 (10.5)	1	10,706	25.8
X	NA	NA	NA	3,435 (8.3)			
NA	NA	X	NA	1,910 (4.6)			
NA	X	NA	NA	991 (2.4)			

*X indicates participation in each survey. JACSIS, Japan COVID-19 and Society Internet Survey (2020, 2021); JASTIS, Japan Society and New Tobacco Internet Survey (2021, 2022); NA, not applicable.

Appendix Table 4. Compliance percentages for each preventive behavior according to participant characteristics*

	3 Cs†				Personal hygiene						Avoid social contact			
Characteristics	Mask-wearing	Ventilation	Social distancing	Avoid crowds	Hand sanitation	Hand washing	Gargling	Respiratory hygiene	Avoid touching face	Surface sanitation	Avoid travel	Avoid going out	Avoid talking closely	Avoid high-risk person
All participants	88.5	40.9	44.3	59.7	66.2	55.5	45.8	73.4	44.5	20.7	72.1	56.4	42.1	59.2
Sex														
M	82.6	33.4	40.2	53.7	59.9	47.3	41.9	64.6	35.6	17.1	65.5	50.7	37.9	51.4
F	94.3	48.3	48.4	65.7	72.6	63.6	49.6	82.2	53.3	24.2	78.6	62.1	46.3	67.0
Age, y														
20–29	78.1	37.0	35.8	48.8	63.8	51.7	46.9	68.8	41.4	27.3	59.4	45.4	32.9	51.4
30–39	85.5	39.6	38.3	56.0	66.5	56.5	49.1	74.9	41.1	23.3	70.2	50.6	34.5	57.4
40–49	89.3	41.3	41.7	58.2	67.0	56.3	48.6	77.6	44.8	23.2	73.4	54.2	38.7	59.0
50–59	91.1	41.0	44.9	59.8	67.0	57.2	44.9	78.0	45.5	19.6	73.6	57.1	43.0	59.8
60–69	92.8	40.2	49.5	62.9	66.3	56.4	40.6	71.9	43.8	15.6	75.0	60.6	47.5	61.8
70–79	92.5	45.9	54.9	71.8	66.5	54.3	44.9	68.1	49.7	15.8	79.5	69.6	55.2	65.2
Education														
Junior high, high school	88.6	38.6	42.9	58.7	65.2	53.4	41.9	70.3	41.3	18.7	73.6	56.2	41.5	58.3
Vocational school, junior college	91.2	46.2	46.1	63.1	71.2	61.8	50.2	79.9	50.5	25.1	75.7	58.5	43.5	63.5
University, graduate school	86.6	41.1	45.3	59.2	64.8	54.9	49.3	74.4	45.5	20.9	67.6	55.4	42.0	57.8
Other	79.5	39.4	47.1	58.5	62.9	46.9	37.4	64.3	46.6	19.7	63.4	56.9	45.4	58.6
Equivalent income, million JPY														
<2.00	87.0	41.6	45.4	61.3	61.3	52.0	42.2	68.1	42.0	18.8	72.6	59.4	44.1	58.2
2.0–3.99	89.1	39.3	42.3	59.1	66.1	54.1	45.1	73.2	43.1	19.3	73.5	56.1	40.2	58.5
4.00–5.99	88.8	38.9	42.4	57.9	68.0	55.1	47.4	76.8	43.5	21.0	68.1	51.9	38.4	57.9
≥6.00	86.2	40.1	43.8	54.5	68.9	55.3	46.9	75.7	46.6	23.9	63.4	51.3	40.9	57.7
Not answered	89.3	44.6	48.0	62.8	68.3	60.8	48.3	74.9	48.4	23.0	75.5	59.6	46.5	62.6
Population density of residential prefecture														
High, top 20%	87.0	43.9	45.8	58.2	66.1	56.1	50.9	72.2	47.3	21.6	68.8	53.9	43.1	58.6
Low, <80%	89.0	39.6	43.7	60.3	66.3	55.2	43.6	73.9	43.3	20.3	73.5	57.4	41.7	59.5

*Values are % compliance. Total number of responses was 103,312. JACSIS, Japan COVID-19 and Society Internet Survey; JASTIS, Japan Society and New Tobacco Internet Survey; JPY, Japanese Yen.
†3 Cs is the government of Japan campaign recommending that the public avoids closed spaces, crowded places, and close-contact settings to prevent COVID-19.

Appendix Table 5. Associations between 3 Cs behavior and participant characteristics*

	3 Cs†							
	Mask-wearing		Ventilation		Social distancing		Avoiding crowds	
Characteristics	% Difference (95% CI)	p value	% Difference (95% CI)	p value	% Difference (95% CI)	p value	% Difference (95% CI)	p value
Per wave‡	3.1 (2.6–3.5)	<0.001	0.2 (–0.3 to 0.8)	0.358	1.4 (0.8 to 1.9)	<0.001	0.4 (–0.1 to 0.9)	0.128
Survey								
JACSIS	Referent	NA	Referent	NA	Referent	NA	Referent	NA
JASTIS	–1.3 (–2.0 to –0.6)	<0.001	–13.4 (–14.4 to –12.3)	<0.001	–1.5 (–2.5 to –0.4)	0.006	–3.2 (–4.3 to –2.1)	<0.001
Sex								
M	Referent	NA	Referent	NA	Referent	NA	Referent	NA

	3 Cs†							
	Mask-wearing		Ventilation		Social distancing		Avoiding crowds	
Characteristics	% Difference (95% CI)	p value	% Difference (95% CI)	p value	% Difference (95% CI)	p value	% Difference (95% CI)	p value
F	11.9 (10.7–13.2)	<0.001	14.4 (12.8–16.0)	<0.001	8.0 (6.4–9.5)	<0.001	11.4 (9.9–12.9)	<0.001
Age, y								
20–29	Referent	NA	Referent	NA	Referent	NA	Referent	NA
30–39	8.1 (5.1–11.0)	<0.001	2.9 (0.3–5.5)	0.031	4.2 (1.7–6.6)	0.001	7.8 (5.1–10.4)	<0.001
40–49	11.4 (8.4–14.4)	<0.001	4.0 (1.4–6.5)	0.002	7.3 (5.0–9.7)	<0.001	9.8 (7.2–12.4)	<0.001
50–59	13.3 (10.2–16.4)	<0.001	4.4 (1.8–7.0)	0.001	10.4 (8.0–12.9)	<0.001	11.3 (8.6–14.0)	<0.001
60–69	15.3 (12.4–18.2)	<0.001	3.8 (1.1–6.4)	0.005	15.7 (13.1–18.2)	<0.001	14.6 (11.9–17.4)	<0.001
70–79	14.5 (11.2–17.8)	<0.001	8.6 (4.8–12.4)	<0.001	20.2 (16.7–23.7)	<0.001	22.3 (19.1–25.5)	<0.001
Education								
Junior high, high school	Referent	NA	Referent	NA	Referent	NA	Referent	NA
Vocational school, junior college	1.2 (0.2–2.3)	0.016	4.9 (3.3–6.4)	<0.001	3.2 (1.6–4.8)	<0.001	3.6 (2.2–5.1)	<0.001
University, graduate school	0.2 (–1.7 to 2.3)	0.826	2.8 (0.7–4.9)	0.008	3.5 (1.6–5.4)	<0.001	2.5 (0.8–4.2)	0.005
Other	–5.3 (–10.6 to –0.05)	0.048	1.1 (–6.1 to 8.2)	0.769	1.9 (–5.5 to 9.2)	0.619	0.0 (–6.7 to 6.7)	0.999
Equivalent income, million JPY								
<2.00	Referent	NA	Referent	NA	Referent	NA	Referent	NA
2.00–3.99	2.8 (1.4–4.2)	<0.001	–1.4 (–3.4 to 0.6)	0.164	–1.8 (–3.8 to 0.2)	0.077	–0.8 (–2.7 to 1.2)	0.444
4.00–5.99	4.3 (2.8–5.9)	<0.001	–0.4 (–2.6 to 1.7)	0.687	0.0 (–2.2 to 2.2)	0.987	0.5 (–1.8 to 2.8)	0.667
>6.00	1.7 (–2.0 to 5.4)	0.358	–1.1 (–4.4 to 2.2)	0.522	0.6 (–2.6 to 3.8)	0.710	–3.7 (–7.7 to 0.3)	0.072
Not answered	1.5 (0.1–2.9)	0.032	1.0 (–1.2 to 3.2)	0.364	2.6 (0.4–4.8)	0.022	1.3 (–0.9 to 3.5)	0.240
Population density of residential prefecture								
High, top 20	Referent	NA	Referent	NA	Referent	NA	Referent	NA
Low, <80%	–1.1 (–2.7 to 0.5)	0.176	4.9 (3.3–6.5)	<0.001	2.7 (1.0–4.8)	0.001	–1.5 (–3.0 to 0.1)	0.065

*Total number of responses was 103,312. JACSIS, Japan COVID-19 and Society Internet Survey; JASTIS, Japan Society and New Tobacco Internet Survey; JPY, Japanese Yen; NA, not applicable.
†3 Cs is the government of Japan campaign recommending that the public avoids closed spaces, crowded places, and close-contact settings to prevent COVID-19.
‡Surveys were conducted in 4 waves: August 25–September 30, 2020 (JACSIS2020); February 8–26, 2021 (JASTIS2021); September 27–October 29, 2021 (JACSIS2021); and February 1–28, 2022 (JASTIS2022).

Appendix Table 6. Associations between personal hygiene behaviors and participant characteristics*

	Personal hygiene											
	Hand sanitation		Hand washing		Gargling		Respiratory hygiene		Avoid touching face		Surface sanitation	
Characteristics	Diff. (95% CI)	p value	Diff. (95% CI)	p value	Diff. (95% CI)	p value	Diff. (95% CI)	p value	Diff. (95% CI)	p value	Diff. (95% CI)	p value
Per wave†	3.6 (3.2–4.1)	<0.001	0.9 (0.3–1.4)	0.001	–0.4 (–0.8 to 0.1)	0.091	3.8 (3.3–4.3)	<0.001	1.3 (0.8–1.9)	<0.001	–0.7 (–1.1 to –0.3)	0.001
Survey												
JACSIS	Referent	NA	Referent	NA	Referent	NA	Referent	NA	Referent	NA	Referent	NA
JASTIS	–0.2 (–1.0 to 0.6)	0.633	–3.5 (-4.4 to –2.6)	<0.001	2.4 (1.6–3.2)	<0.001	–2.4 (–3.4 to –1.4)	<0.001	–1.0 (–2.0 to –0.02)	0.046	0.6 (–0.1 to 1.3)	0.072
Sex												
M	Referent	NA	Referent	NA	Referent	NA	Referent	NA	Referent	NA	Referent	NA
F	12.3 (10.6–14.0)	<0.001	16.1 (14.3–17.8)	<0.001	7.8 (5.9–9.7)	<0.001	18.2 (16.6–19.7)	<0.001	17.1 (15.5–18.7)	<0.001	6.5 (5.0–8.0)	<0.001
Age, y												
20–29	Referent	NA	Referent	NA	Referent	NA	Referent	NA	Referent	NA	Referent	NA

	Personal hygiene											
	Hand sanitation		Hand washing		Gargling		Respiratory hygiene		Avoid touching face		Surface sanitation	
Characteristics	Diff. (95% CI)	p value	Diff. (95% CI)	p value	Diff. (95% CI)	p value	Diff. (95% CI)	p value	Diff. (95% CI)	p value	Diff. (95% CI)	p value
30–39	3.5 (0.1–6.9)	0.043	5.9 (2.8–9.0)	<0.001	4.3 (1.1–7.6)	0.009	7.7 (4.9–10.6)	<0.001	1.8 (–1.0 to 4.6)	0.213	–3.6 (–6.1 to –1.1)	0.005
40–49	3.0 (–0.1 to 6.1)	0.060	4.8 (1.8–7.8)	0.002	3.0 (–0.1 to 6.0)	0.058	9.8 (6.9–12.7)	<0.001	4.6 (1.9–7.4)	0.001	–4.2 (–6.8 to –1.7)	0.001
50–59	2.9 (–0.2 to 6.1)	0.069	5.5 (2.4–8.6)	<0.001	0.5 (–2.6 to 3.6)	0.766	9.9 (6.9–12.9)	<0.001	5.4 (2.5–8.3)	<0.001	–7.7 (–10.3 to –5.0)	<0.001
60–69	3.1 (–0.1 to 6.2)	0.054	6.2 (3.1–9.3)	<0.001	–2.3 (–5.5 to 0.9)	0.158	5.6 (2.6–8.5)	<0.001	5.4 (2.5–8.4)	<0.001	–10.9 (–13.4 to –8.4)	<0.001
70–79	4.4 (0.3–8.5)	0.037	3.0 (–1.1 to 7.1)	0.147	0.4 (–3.9 to 4.8)	0.838	0.9 (–2.7 to 4.5)	0.633	10.5 (6.8–14.2)	<0.001	–10.8 (–14.4 to –7.2)	<0.001
Education												
Junior high, high school	Referent	NA	Referent	NA	Referent	NA	Referent	NA	Referent	NA	Referent	NA
Vocational school, junior college	2.9 (1.4–4.4)	<0.001	4.4 (2.7–6.1)	<0.001	5.5 (3.6–7.4)	<0.001	4.2 (2.9–5.6)	<0.001	5.9 (4.2–7.5)	<0.001	3.5 (2.2–4.7)	<0.001
University, graduate school	0.1 (–2.1 to 2.2)	0.943	2.1 (–0.1 to 4.3)	0.067	5.6 (3.2–8.0)	<0.001	5.2 (3.3–7.1)	<0.001	4.2 (2.1–6.4)	<0.001	0.8 (–1.1 to 2.7)	0.427
Other	–0.7 (–7.0 to 5.6)	0.829	–2.1 (–9.6 to 5.3)	0.574	0.2 (–5.7 to 6.1)	0.943	–1.6 (–8.1 to 4.9)	0.626	3.8 (–2.8 to 10.4)	0.262	0.5 (–4.7 to 5.7)	0.847
Equivalent income, million JPY												
<2.00	Referent	NA	Referent	NA	Referent	NA	Referent	NA	Referent	NA	Referent	NA
2.00–3.99	3.7 (1.9–5.5)	<0.001	2.4 (0.4–4.4)	0.019	2.1 (0.1–4.1)	0.043	4.0 (2.3–5.7)	<0.001	1.1 (–1.1 to 3.3)	0.328	0.4 (–1.2 to 2.0)	0.603
4.00–5.99	6.6 (4.6–8.5)	<0.001	3.5 (1.4–5.7)	0.001	3.0 (0.9–5.1)	0.005	7.4 (5.5–9.3)	<0.001	3.3 (0.8–5.8)	0.009	2.0 (0.4–3.7)	0.017
≥6.00	8.3 (4.9–11.7)	<0.001	4.1 (0.4–7.9)	0.029	2.4 (–0.9 to 5.7)	0.159	6.2 (2.5–9.8)	0.001	4.6 (0.1–9.1)	0.047	4.4 (1.0–7.7)	0.010
Not answered	3.9 (2.0–5.9)	<0.001	5.5 (3.3–7.7)	<0.001	3.7 (1.5–6.0)	0.001	3.5 (1.7–5.3)	<0.001	4.0 (1.7–6.3)	0.001	2.8 (1.0–4.5)	0.002
Population density of residential prefecture												
High, top 20%	Referent	NA	Referent	NA	Referent	NA	Referent	NA	Referent	NA	Referent	NA
Low, <80%	0.3 (–1.6 to 2.2)	0.752	2.2 (0.3 to 4.1)	0.024	6.7 (4.7–8.7)	<0.001	–1.8 (–3.6 to –0.03)	0.046	4.8 (3.1–6.5)	<0.001	0.9 (–0.7 to 2.5)	0.284

*Values are % difference (95% CI) unless otherwise noted. Total number of responses was 103,312. Diff., difference; JACSIS, Japan COVID-19 and Society Internet Survey; JASTIS, Japan Society and New Tobacco Internet Survey; JPY, Japanese Yen; NA, not applicable.

†Surveys were conducted in 4 waves: August 25–September 30, 2020 (JACSIS2020); February 8–26, 2021 (JASTIS2021); September 27–October 29, 2021 (JACSIS2021); and February 1–28, 2022 (JASTIS2022).

Appendix Table 7. Associations between social contact behaviors and participant characteristics*

	Avoid social contact							
	Avoid travel		Avoid going out		Avoid talking closely		Avoid high-risk person	
Characteristics	% Difference (95% CI)	p value	% Difference (95% CI)	p value	% Difference (95% CI)]	p value	% Difference (95% CI)	p value
Per wave†	0.3 (–0.2 to 0.8)	0.274	–1.5 (–2.0 to –1.1)	<0.001	1.9 (1.4–2.4)	<0.001	2.3 (1.8–2.8)	<0.001
Survey								
JACSIS	Referent	NA	Referent	NA	Referent	NA	Referent	NA
JASTIS	–1.5 (–2.4 to –0.6)	0.001	–2.0 (–2.9 to –1.0]	<0.001	–2.0 (–3.0 to –1.0]	<0.001	–6.0 (–7.0 to –5.0)	<0.001
Sex								
M	Referent	NA	Referent	NA	Referent	NA	Referent	NA
F	12.0 (10.5–13.4)	<0.001	10.4 (8.9–12.0)	<0.001	7.6 (6.0–9.2)	<0.001	15.3 (13.7–16.8)	<0.001
Age, y								
20–29	Referent	NA	Referent	NA	Referent	NA	Referent	NA
30–39	10.9 (8.2–13.7)	<0.001	4.8 (2.0–7.5)	0.001	2.5 (–0.1 to 5.0)	0.062	6.4 (3.6–9.2)	<0.001
40–49	13.6 (10.8–16.3)	<0.001	8.4 (5.7–11.1)	<0.001	6.9 (4.3–9.5)	<0.001	7.5 (4.7–10.3)	<0.001
50–59	14.3 (11.4–17.1)	<0.001	11.2 (8.4–14.0)	<0.001	10.9 (8.2–13.7)	<0.001	8.3 (5.4–11.2)	<0.001
60–69	14.5 (11.7–17.3)	<0.001	15.0 (12.1–17.8)	<0.001	15.6 (12.9–18.4)	<0.001	10.7 (7.9–13.6)	<0.001
70–79	19.1 (15.5–22.6)	<0.001	22.8 (19.2–26.3)	<0.001	23.1 (19.3–26.9)	<0.001	13.1 (9.4–16.8)	<0.001
Education								
Junior high, high school	Referent	NA	Referent	NA	Referent	NA	Referent	NA
Vocational school, junior college	0.5 (–0.8 to 1.9)	0.483	2.4 (0.9–3.9)	0.002	2.6 (1.2–4.1)	<0.001	2.5 (1.1–3.9)	0.001
University, graduate-school	–3.3 (–5.2 to –1.4)	0.001	2.1 (0.2–4.0)	0.029	2.7 (0.7–4.7)	0.008	0.6 (–1.4 to 2.6)	0.550
Other	–6.6 (–13.9 to 0.6)	0.074	2.2 (–5.1 to 9.4)	0.560	2.3 (–5.1 to 9.7)	0.541	0.9 (–6.8 to 8.6)	0.825
Equivalent income, million JPY								
<2.00	Referent	NA	Referent	NA	Referent	NA	Referent	NA
2.00–3.99	1.5 (–0.1 to 3.2)	0.066	–1.3 (–3.0 to 0.5)	0.149	–2.5 (–4.4 to –0.6)	0.012	1.4 (–0.5 to 3.2)	0.155
4.00–5.99	–0.6 (–2.6 to 1.3)	0.518	–2.1 (–4.2 to –0.1)	0.044	–1.7 (–3.8 to 0.5)	0.131	2.9 (0.8–5.1)	0.006
≥6.00	–5.1 (–8.6 to –1.5)	0.005	–3.5 (–7.0 to 0.02)	0.051	–0.1 (–3.7 to 3.6)	0.972	2.5 (–1.1 to 6.2)	0.171
Not answered	2.0 (0.1–3.8)	0.039	0.8 (–1.2 to 2.8)	0.440	2.4 (0.2–4.6)	0.033	3.0 (0.9–5.1)	0.005
Population density of residential prefecture								
High, top 20%	Referent	NA	Referent	NA	Referent	NA	Referent	NA
Low, < 80%	–2.5 (–4.2 to –0.9)	0.003	–2.8 (–4.5 to –1.1)	0.001	1.9 (0.1–3.6)	0.035	–0.1 (–1.9 to 1.6)	0.896

*Total number of responses was 103,312. JACSIS, Japan COVID-19 and Society Internet Survey; JASTIS, Japan Society and New Tobacco Internet Survey; JPY, Japanese yen; NA, not applicable.
†Surveys were conducted in 4 waves: August 25–September 30, 2020 (JACSIS2020); February 8–26, 2021 (JASTIS2021); September 27–October 29, 2021 (JACSIS2021); and February 1–28, 2022 (JASTIS2022).

Appendix Table 8. Percentage of survey participants who were compliant with each COVID-19 preventive behavior according to latent class*

Class	Mask wearing	Ventilation	Social distancing	Avoid crowds	Hand sanitation	Hand washing	Gargling	Respiratory hygiene	Avoid touching face	Surface sanitation	Avoid travel	Avoid going out	Avoid talking closely	Avoid high-risk person
Low compliance	49.4	6.7	4.8	9.4	16.2	8.6	8.5	16.8	3.0	1.1	29.4	12.7	5.3	7.8
Personal hygiene	96.2	34.2	15.1	17.4	78.8	62.4	52.2	88.3	38.3	11.8	55.4	21.3	15.9	28.7
Avoid social contact	97.1	30.3	53.9	92.5	63.2	48.8	33.0	76.6	34.2	2.3	92.1	81.4	50.5	82.6
Comprehensive	99.4	86.2	88.9	95.2	95.7	92.6	84.0	97.7	94.5	67.2	94.1	90.1	83.5	96.4
All participants	88.4	40.9	44.3	59.7	66.2	55.5	45.8	73.4	44.5	20.7	72.1	56.4	42.1	59.2

*Values are % compliance. Total number of responses was 103,312.

Appendix Table 9. Characteristics of participants among each identified latent class*

Characteristic	Low compliance	Personal hygiene	Avoid social contact	Comprehensive	All participants
No. participants	19,509	24,445	33,282	26,076	103,312
Sex					
M	13,858 (71.0)	11,813 (48.3)	15,731 (47.3)	10,139 (38.9)	51,540 (49.9)
F	5,651 (29.0)	12,632 (51.7)	17,551 (52.7)	15,937 (61.1)	51,772 (50.1)
Age, y					
20–29	4,374 (22.4)	4,236 (17.3)	3,232 (9.7)	3,808 (14.6)	15,650 (15.1)
30–39	3,188 (16.4)	3,878 (15.9)	4,199 (12.6)	3,893 (14.9)	15,158 (14.7)
40–49	3,684 (18.9)	5,183 (21.2)	5,862 (17.6)	5,422 (20.8)	20,151 (19.5)
50–59	3,043 (15.6)	4,502 (18.4)	5,840 (17.6)	4,543 (17.5)	17,928 (17.3)
60–69	2,933 (15.0)	3,995 (16.4)	6,923 (20.8)	4,182 (16.0)	18,033 (17.5)
70–79	2,287 (11.7)	2,651 (10.8)	7,226 (21.7)	4,228 (16.2)	16,392 (15.9)
Education					
Junior high school, high school	10,119 (51.9)	11,719 (47.9)	17,139 (51.5)	11,420 (43.8)	50,398 (48.8)
Vocational school, junior college	2,905 (14.9)	5,180 (21.2)	6,490 (19.5)	6,244 (24.0)	20,820 (20.2)
University, graduate-school	6,300 (32.3)	7,402 (30.3)	9,418 (28.3)	8,222 (31.5)	31,341 (30.3)
Other	185 (0.9)	144 (0.6)	235 (0.7)	190 (0.7)	753 (0.7)
Equivalent income, million JPY					
<2.00	3,815 (19.6)	3,651 (14.9)	6,528 (19.6)	4,267 (16.4)	18,261 (17.7)
2.00 to 3.99	7,040 (36.1)	9,339 (38.2)	12,569 (37.8)	9,028 (34.6)	37,976 (36.8)
4.00 to 5.99	2,737 (14.0)	3,796 (15.5)	4,224 (12.7)	3,548 (13.6)	14,305 (13.8)
≥6.00	2,031 (10.4)	2,710 (11.1)	2,432 (7.3)	2,567 (9.9)	9,741 (9.4)
Not answered	3,886 (19.9)	4,949 (20.3)	7,529 (22.6)	6,666 (25.5)	23,029 (22.3)
Population density of residential prefecture					
Low	13,502 (69.2)	16,884 (69.1)	24,585 (73.9)	17,762 (68.1)	72,732 (70.4)
High	6,007 (30.8)	7,561 (30.9)	8,697 (26.1)	8,314 (31.9)	30,580 (29.6)

*Values are no. (%). Total number of survey responses was 103,313. JACSIS, Japan COVID-19 and Society Internet Survey; JASTIS, Japan Society and New Tobacco Internet Survey; JPY, Japanese yen.

Appendix Table 10. Odds ratios measuring associations between each latent class and participant characteristics*

	Latent class					
	Personal hygiene		Avoiding social contact		Comprehensive	
Characteristic	OR (95% CI)	p value	OR (95% CI)	p value	OR (95% CI)	p value
Per wave†	1.31 (1.26–1.36)	<0.001	1.19 (1.15–1.23)	<0.001	1.17 (1.12–1.21)	<0.001
Survey						
JACSIS	Referent		Referent		Referent	
JASTIS	0.82 (0.76–0.86)	<0.001	0.78 (0.73–0.84)	<0.001	0.77 (0.72–0.82)	<0.001
Sex						
M	Referent		Referent		Referent	
F	2.73 (2.49–2.98)	<0.001	2.75 (2.50–3.02)	<0.001	3.88 (3.49–4.31)	<0.001
Age, y						
20–29	Referent		Referent		Referent	
30–39	1.35 (1.14–1.60)	0.001	1.87 (1.59–2.19)	<0.001	1.54 (1.30–1.82)	<0.001
40–49	1.57 (1.33–1.89)	<0.001	2.30 (1.97–2.68)	<0.001	1.87 (1.59–2.21)	<0.001
50–59	1.68 (1.40–2.02)	<0.001	2.84 (2.42–3.22)	<0.001	1.93 (1.62–2.30)	<0.001
60–69	1.65 (1.38–1.98)	<0.001	3.51 (2.97–4.14)	<0.001	1.98 (1.65–2.38)	<0.001
70–79	1.42 (1.16–1.74)	0.001	4.74 (3.87–5.80)	<0.001	2.53 (1.99–3.22)	<0.001
Education						
Junior high school, high school	Referent		Referent		Referent	
Vocational school, junior college	1.28 (1.15–1.41)	<0.001	1.25 (1.13–1.38)	<0.001	1.54 (1.38–1.71)	<0.001
University, graduate school	1.12 (1.004–1.24)	0.043	1.11 (0.99–1.24)	0.070	1.34 (1.17–1.52)	<0.001
Other	0.74 (0.44–1.24)	0.253	0.81 (0.52–1.31)	0.393	1.00 (0.66–1.54)	0.982
Equivalent income, million JPY						
<2.00	Referent		Referent		Referent	
2.00 to 3.99	1.44 (1.27–1.64)	<0.001	1.16 (1.03–1.32)	0.016	1.21 (1.04–1.41)	0.013
4.00 to 5.99	1.58 (1.37–1.83)	<0.001	1.19 (1.03–1.37)	0.019	1.36 (1.16–1.60)	<0.001
≤6.00	1.59 (1.25–2.01)	<0.001	0.96 (0.79–1.17)	0.700	1.36 (1.09–1.71)	0.007
Not answered	1.18 (1.03–1.35)	0.014	1.10 (0.95–1.27)	0.189	1.35 (1.15–1.58)	<0.001
Population density of residential prefecture						
Low	Referent		Referent		Referent	
High	1.07 (0.97–1.18)	0.150	0.89 (0.81–0.98)	0.015	1.15 (1.03–1.28)	0.012

*Referent was low compliance. Total number of survey responses was 103,312. JACSIS, Japan COVID-19 and Society Internet Survey; JASTIS, Japan Society and New Tobacco Internet Survey; JPY, Japanese yen; OR, odds ratio.
†Surveys were conducted in 4 waves: August 25–September 30, 2020 (JACSIS2020); February 8–26, 2021 (JASTIS2021); September 27–October 29, 2021 (JACSIS2021); and February 1–28, 2022 (JASTIS2022).

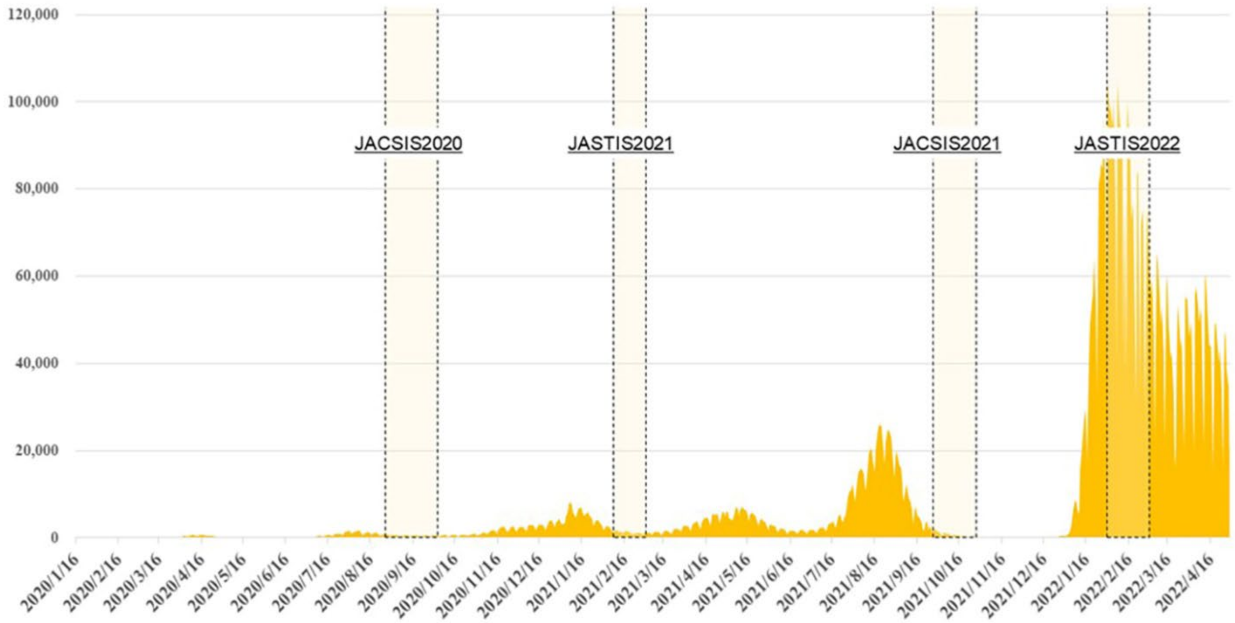

Appendix Figure 1. Daily incidence of COVID-19 in Japan and survey periods of this study. Four surveys were conducted during August 25–September 30, 2020 (JACSIS2020); February 8–26, 2021 (JASTIS2021); September 27–October 29, 2021 (JACSIS2021); and February 1–28, 2022 (JASTIS2022). JACSIS, Japan COVID-19 and Society Internet Survey; JASTIS, Japan Society and New Tobacco Internet Survey. Data were obtained from https://www.mhlw.go.jp/stf/covid-19/open-data.html.

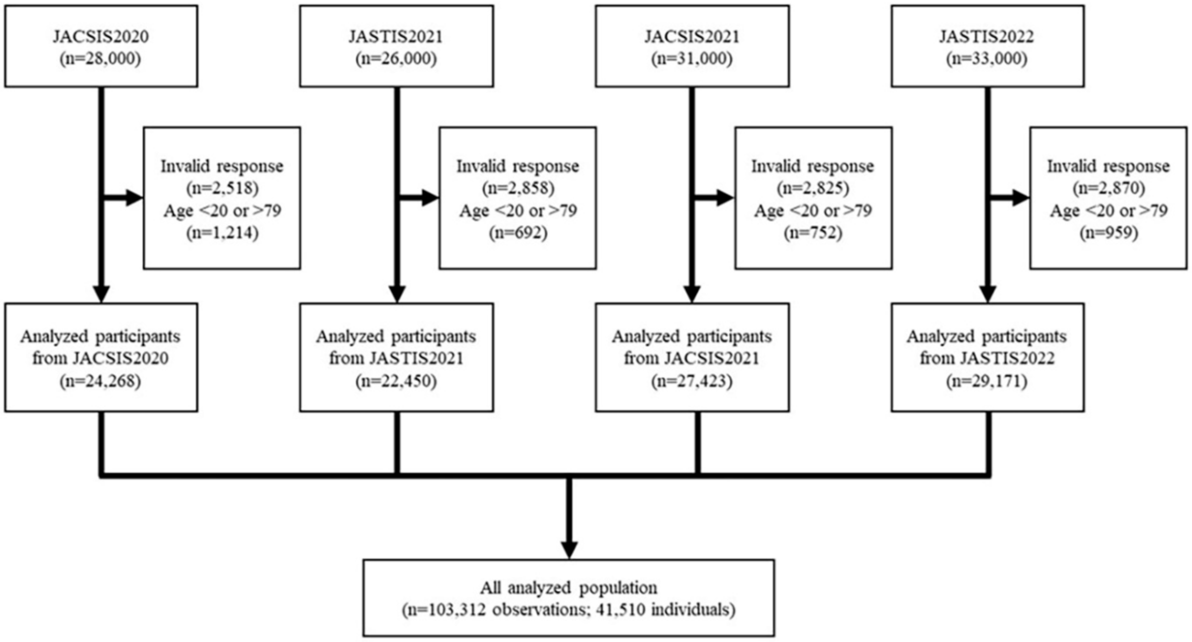

Appendix Figure 2. Flow diagram of inclusion/exclusion criteria for survey participants. Four surveys were conducted during August 25–September 30, 2020 (JACSIS2020); February 8–26, 2021 (JASTIS2021); September 27–October 29, 2021 (JACSIS2021); and February 1–28, 2022 (JASTIS2022). JACSIS, Japan COVID-19 and Society Internet Survey; JASTIS, Japan Society and New Tobacco Internet Survey.

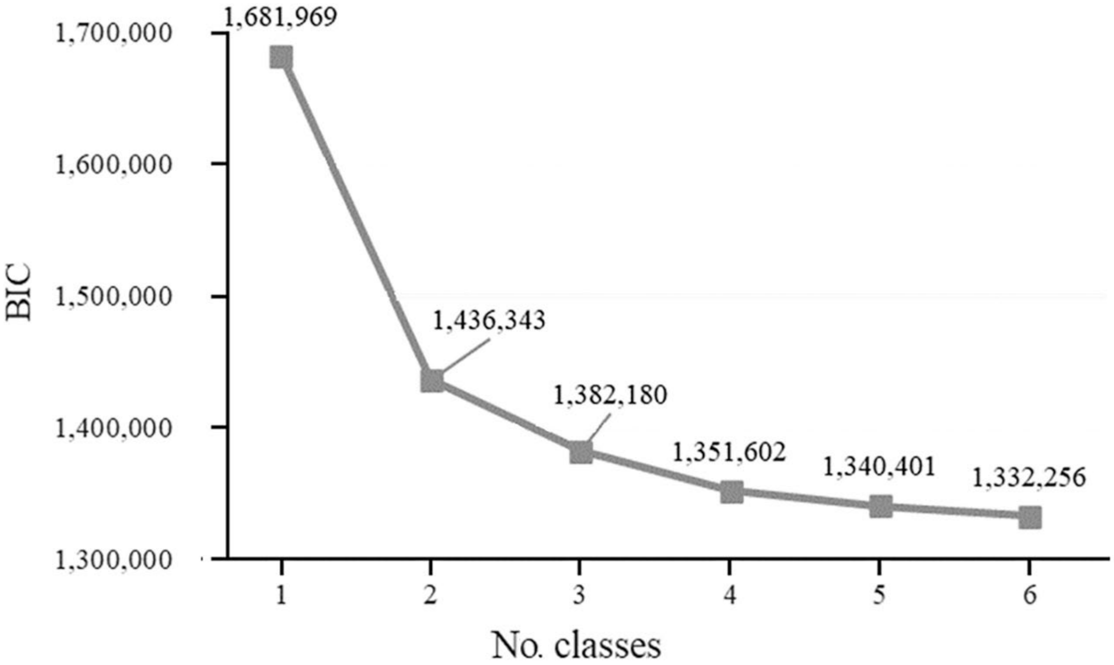

Appendix Figure 3. Latent class analysis of COVID-19 preventive behaviors. Scree plot of Bayesian information criterion (BIC) in each model and different numbers of classes (from 1 to 6) were used to define the number of latent classes evaluated in this study.
